# One-Dimensional Thermal Analysis of the Flexible Electronic Devices Integrated with Human Skin

**DOI:** 10.3390/mi7110210

**Published:** 2016-11-18

**Authors:** Yun Cui, Yuhang Li, Yufeng Xing, Tianzhi Yang, Jizhou Song

**Affiliations:** 1 Institute of Solid Mechanics, Beihang University (BUAA), Beijing 100191, China; kyalex@sina.com (Y.C.); xingyf@buaa.edu.cn (Y.X.); 2 Key Laboratory of Soft Machines and Smart Devices of Zhejiang Province, Zhejiang University, Hangzhou 310027, China; 3 State Key Laboratory of Digital Manufacturing Equipment and Technology, Huazhong University of Science and Technology, Wuhan 430074, China; 4 School of Aerospace Engineering, Shenyang Aerospace University, Shenyang 110136, China; yangtz@sau.edu.cn; 5 Department of Engineering Mechanics and Soft Matter Research Center, Zhejiang University, Hangzhou 310027, China

**Keywords:** flexible electronics, thermal analysis, human skin

## Abstract

A one-dimensional analytic thermal model for the flexible electronic devices integrated with human skin under a constant and pulsed power is developed. The Fourier heat conduction equation is adopted for the flexible electronics devices while the Pennes bio-heat transfer equation is adopted for the skin tissue. Finite element analysis is performed to validate the analytic model through the comparison of temperature distributions in the system. The influences of geometric and loading parameters on the temperature increase under a pulsed power are investigated. It is shown that a small duty cycle can reduce the temperature increase of the system effectively. A thin substrate can reduce the device temperature but increase the skin surface temperature. The results presented may be helpful to optimize the design of flexible electronic devices to reduce the adverse thermal influences in bio-integrated applications.

## 1. Introduction

Recent advances in flexible electronics enable the development of epidermal electronics [1,2], which could be mounted onto the skin and retain conformal contact with the skin under compression and tension. Knowledge on heat transfer within skin tissue due to the physical coupling of epidermal electronics to the skin is critical for the use of epidermal electronics since even only a few degrees in temperature increase may induce uncomfortable feelings.

Many researchers have performed the thermal analysis of flexible electronic devices by adopting the Fourier heat conduction equation. Kim et al. [3], Lu et al. [4], and Cui et al. [5] studied the heat conduction of flexible micro-scale inorganic light-emitting diodes under a constant power experimentally and analytically. Relevant experimental results are presented as surface temperature contours, which can be captured by QFI Infra-Scope Micro-Thermal Imager [3]. Results of experiment agree well with analytic ones. Kim et al. [6] and Li et al. [7] investigated the thermal properties of flexible devices on various substrates under a pulsed power. They developed an analytic axi-symmetric model to explore the dependence of device temperature on the geometric dimensions, material properties and loading parameters. There exist several reviews of thermal and mechanical analysis of flexible electronics [8,9].

**Figure 1:**
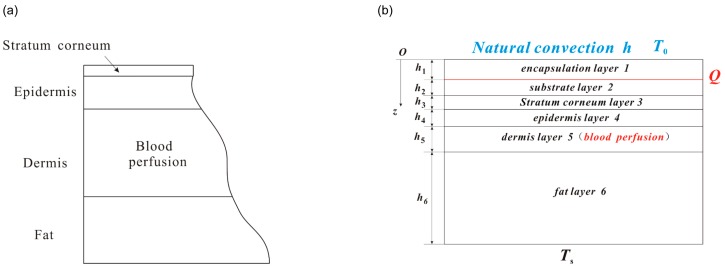
(**a**) Schematic illustration of the skin tissue structure; and (**b**) schematic illustration of one-dimensional geometry of the analytic modeled device-skin system.

The heat transfer in skin tissue is much more complicated than that in flexible electronic devices due to the influences of blood perfusion and metabolism and the complex multilayered structure consisting of four layers: stratum corneum, epidermis, dermis and fat as shown in Figure 1a. The Pennes bioheat equation has been widely used to investigate the thermal response of skin tissue under surface heating. Jiang et al. [10] studied the skin burn process resulting from a high temperature heat source to the skin surface by using finite difference method to solve the Pennes bio-heat equation. Ozen et al. [11] presented a one-dimensional multi-layer model to characterize the temperature rise resulting from skin exposure to microwaves. Im et al. [12] carried out a numerical study on the temperature profiles as a result of local heating of human skin. An extensive review on heat transfer of skin tissue was given by Xu et al. [13].

The above heat transfer analyses are either for flexible electronic devices or human skin. There are few studies on the system of flexible electronic devices integrated with the human skin. For better understanding of the thermal properties of device and establishing design guidelines for flexible electronic devices to minimize the adverse thermal effect, we aims to develop an analytical model for the flexible electronic devices integrated with human skin under both constant and pulsed power. Basing on the Kim’s experimental results and analytical model for the device on top of a metal trunk [3,4], this paper aims at the device integrated with human skin. For flexible electronic devices with the in-plane dimension on the order of a few millimeters, which is much larger than the device thickness, the heat mainly transfers along the thickness direction. It is reasonable to perform a one-dimensional thermal analysis. Such treatment simplifies the theoretical model significantly with enough accuracy. Moreover, the finite element models are also established to validate the analytic solutions.

## 2. Thermal Analysis under a Constant Power

The human skin is modeled as a multi-layer structure consisting of four layers: stratum corneum, epidermis, dermis and fat. The flexible electronic device consists of the functional component on a flexible substrate (e.g., polydimethylsiloxane (PDMS)) encapsulated by an encapsulation layer (e.g., SU8). The functional component could be modeled as a planar heat source with heat generation power density *Q* (W/m^2^) since its thermal conductivity (~100 W/m/K) is much larger than that of substrate or encapsulation layer. Figure 1b shows the one-dimensional geometry of the analytic model with the flexible device on the human skin. The origin of the coordinate (*z*) is established on the top surface of encapsulation layer with the positive direction pointing from the flexible device to the skin tissue. The total thickness of the system is denoted by *H*. *h_i_* denotes the thickness for each layer with the substrate *i* (*i* = 1, 2, 3, 4, 5 and 6) for the encapsulation, substrate, stratum corneum, epidermis, dermis and fat layer, respectively. The top surface of encapsulation layer has the natural convection boundary with *h* as the coefficient of heat convection. The bottom surface of fat layer has constant core body temperature *T*_s_.

The temperature in the device satisfies the Fourier heat conduction equation

(1) $$k_{i}\frac{d^{2}T_{i}}{dz^{2}}=0\;\;\;\;\;\;\;\;\;\;\left(i=1,2\right)$$

while the temperature in the skin tissue satisfies the Pennes bio-heat equation [14]

(2) $$\left\{ \begin{array}{l} k_{i}\frac{d^{2}T_{i}}{dz^{2}}+q_{\mathrm{met}}=0\;\;\;\;\;\;\;\;\;\;\left(i=3,4,6\right)\\ k_{5}\frac{d^{2}T_{5}}{dz^{2}}-\varpi_{b}\rho_{b}c_{b}\left(T_{5}-T_{s}\right)+q_{\mathrm{met}}=0 \end{array}\right.$$

where *k_i_* represents the thermal conductivity of corresponding layers; ρ*_b_* and *c_b_* are the density and specific heat of blood, respectively; $\varpi_{b}$ is the blood perfusion rate; and *q*_met_ is the metabolic heat generation. Here, we assumed that the blood temperature is just the same as the core temperature [14]. The main difference between two heat conduction models mentioned above is confined to blood perfusion effect and metabolism. The blood perfusion is effected by $\varpi_{b}$, which varies from 0–1 mL/(mL·s) [15]. While the metabolic heat generation is almost constant for healthy person. It should be noted that the blood perfusion only exists inside the dermis layer [13].

Let Δ*T* = *T*(*z*) − *T*_s_ denote the temperature increase from the core temperature. Equations (1) and (2) then become

(3) $$\left\{ \begin{array}{l} k_{i}\frac{d^{2}\Delta T_{i}}{dz^{2}}=0\;\;\;\;\;\;\;\;\;\;\left(i=1,2\right)\\ k_{i}\frac{d^{2}\Delta T_{i}}{dz^{2}}+q_{\mathrm{met}}=0\;\;\;\;\;\;\;\;\;\;\left(i=3,4,6\right)\\ k_{5}\frac{d^{2}\Delta T_{5}}{dz^{2}}-\varpi_{b}\rho_{b}c_{b}\Delta T_{5}+q_{\mathrm{met}}=0 \end{array}\right.\;\;\;\;$$

The temperature increase in each layer can be given

(4) $$\left\{ \begin{array}{l} \Delta T_{1}=A_{1}z+B_{1}\\ \Delta T_{2}=A_{2}z+B_{2}\\ \Delta T_{3}=-q_{\mathrm{met}}z^{2}/2k_{3}+B_{3}z+C_{3}\\ \Delta T_{4}=-q_{\mathrm{met}}z^{2}/2k_{4}+B_{4}z+C_{4}\\ \Delta T_{5}=A_{5}\exp\left(z\sqrt{\eta /k_{5}}\right)+B_{5}\exp\left(-z\sqrt{\eta /k_{5}}\right)+q_{\mathrm{met}}/\eta \\ \Delta T_{6}=-q_{\mathrm{met}}z^{2}/2k_{6}+B_{6}z+C_{6} \end{array}\right.\;$$

where $\eta =\varpi_{b}\rho_{b}c_{b}$, and the coefficients *A_i_*, *B_i_* and *C_i_* are to be determined by the boundary conditions and the interfacial continuity condition. At the top surface of encapsulation layer (*z* = 0), the natural convection condition gives

(5) $${-k_{1}\frac{d\Delta T}{dz}|}_{z=0}=-h\cdot {\left(\Delta T-\Delta T_{0}\right)|}_{z=0}$$

where Δ*T*_0_ = *T*_0_ − *T*_s_, *T*_0_ denotes the ambient temperature, *k*_1_ is the thermal conductivity of encapsulation layer and *h* is the coefficient of heat convection. At the encapsulation/substrate interface (*z* = *h*_1_), the temperature is continuous while the heat flux satisfies the surface heat source condition, which gives,

(6) $${\Delta T|}_{z=h_{1}^{+}}={\Delta T|}_{z=h_{1}^{-}}\;\text{and}\;k_{1}{\frac{d\Delta T}{dz}|}_{z=h_{1}^{-}}-k_{2}{\frac{d\Delta T}{dz}|}_{z=h_{1}^{+}}=Q$$

where *k*_2_ is the thermal conductivity of substrate layer. At the interface between any other two layers, both the temperature and heat flux are continuous, which give

(7) $${\Delta T|}_{z={\left(\sum_{i=1}^{n}h_{i}\right)}^{+}}={\Delta T|}_{z={\left(\sum_{i=1}^{n}h_{i}\right)}^{-}}\;\text{and}\;k_{\left(n+1\right)}{\frac{d\Delta T}{dz}|}_{z={\left(\sum_{i=1}^{n}h_{i}\right)}^{+}}=k_{n}{\frac{d\Delta T}{dz}|}_{z={\left(\sum_{i=1}^{n}h_{i}\right)}^{-}}\;\;$$

where *n* = 2, 3, 4, and *k_n_* is the thermal conductivity of layer *n* with *n* = 2 for substrate *n* = 3 for stratum corneum, *n* = 4 for epidermis, and *n* = 5 for dermis.

The ambient temperature at the bottom surface ($z=\sum_{i=1}^{6}h_{i}$) of fat layer gives

(8) $${\Delta T|}_{z=\sum_{i=1}^{6}h_{i}}=0$$

It should be noted that the stratum corneum usually has the same thermal property as that of epidermis, i.e., *k*_3_ = *k*_4_. The coefficients in Equation (4) are then determined by the boundary and continuity conditions in Equations (5)–(8) as

(9) $$\left\{\begin{array}{l} A_{1}\\ B_{1}\\ A_{2}\\ B_{2}\\ B_{3}\\ C_{3}\\ B_{4}\\ C_{4}\\ A_{5}\\ B_{5}\\ B_{6}\\ C_{6} \end{array}\right\}=\left\{\begin{array}{l} \frac{y\left[-E\sin h(y)+F\cos h(y)\right]-E\cos h(y)+F\sin h(y)-M}{y\left[C\sin h(y)+D\cos h(y)\right]+C\cos h(y)+D\sin h(y)}\\ \frac{k_{1}}{h}A_{1}+\Delta T_{0}\\ \left(k_{1}A_{1}-Q\right)/k_{2}\\ h_{1}A_{1}+B_{1}-h_{1}A_{2}\\ \left[k_{1}A_{1}-Q+q_{\mathrm{met}}\left(h_{1}+h_{2}\right)\right]/k_{3}\\ A_{2}\left(h_{1}+h_{2}\right)+h_{1}A_{1}+B_{1}-h_{1}A_{2}+q_{\mathrm{met}}{\left(h_{1}+h_{2}\right)}^{2}/\left(2k_{3}\right)-B_{3}\left(h_{1}+h_{2}\right)\\ \left[k_{1}A_{1}-Q+q_{\mathrm{met}}\left(h_{1}+h_{2}\right)\right]/k_{3}\\ A_{2}\left(h_{1}+h_{2}\right)+h_{1}A_{1}+B_{1}-h_{1}A_{2}+q_{\mathrm{met}}{\left(h_{1}+h_{2}\right)}^{2}/\left(2k_{3}\right)-B_{3}\left(h_{1}+h_{2}\right)\\ \left[\left(C+D\right)A_{1}+E-F\right]e^{-\left(h_{1}+h_{2}+h_{3}+h_{4}\right)\sqrt{\eta /k_{5}}}/2\\ \left[\left(C-D\right)A_{1}+E+F\right]e^{\left(h_{1}+h_{2}+h_{3}+h_{4}\right)\sqrt{\eta /k_{5}}}/2\\ \left[\begin{array}{l} y\left(CA_{1}+E\right)\sin h(y)/h_{6}+y(DA_{1}-F)\cos h(y)/h_{6}\\ +q_{\mathrm{met}}\left(h_{1}+h_{2}+h_{3}+h_{4}+h_{5}\right)/k_{6} \end{array}\right]\\ \left[\begin{array}{l} \left(CA_{1}+E\right)\cos h(y)+(DA_{1}-F)\sin h(y)+q_{\mathrm{met}}/\eta \\ +q_{\mathrm{met}}{\left(h_{1}+h_{2}+h_{3}+h_{4}+h_{5}\right)}^{2}/2k_{6}-B_{6}\left(h_{1}+h_{2}+h_{3}+h_{4}+h_{5}\right) \end{array}\right] \end{array}\right\}$$

where,

$$\begin{array}{l} C=h_{1}+k_{1}/h+k_{1}h_{2}/k_{2}+k_{1}\left(h_{3}+h_{4}\right)/k_{3}\\ D=k_{1}/\sqrt{k_{5}\eta }\\ E=-(h_{2}/k_{2}+\left(h_{3}+h_{4}\right)/k_{3})Q-q_{\mathrm{met}}{\left(h_{3}+h_{4}\right)}^{2}/\left(2k_{3}\right)-q_{\mathrm{met}}/\eta +\Delta T_{0}\\ F=\left(Q+q_{\mathrm{met}}\left(h_{3}+h_{4}\right)\right)/\sqrt{k_{5}\eta }\\ M=q_{\mathrm{met}}/\eta -q_{\mathrm{met}}h_{6}^{2}/\left(2k_{6}\right)\\ y=h_{5}\sqrt{\eta /k_{5}} \end{array}$$

**Figure 2:**
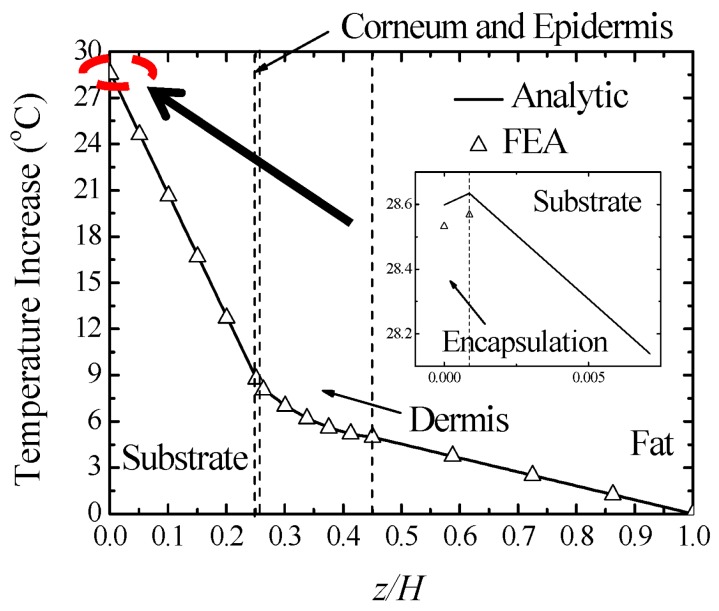
The comparison of temperature increase along the thickness direction between the analytic prediction and finite element analysis with the total thickness *H* = 8.007 mm.

In order to validate the analytical solutions in Equation (9), finite element analysis (FEA) is performed using ABAQUS software (6.13-1, Dassault Simulia, Waltham, MA, USA) to study the thermal properties of this system with continuum element DC3D8. The thicknesses of encapsulation, substrate, stratum corneum, epidermis, dermis, and fat are taken as 7 μm, 2 mm, 0.02 mm, 0.08 mm, 1.5 mm and 4.4 mm, respectively [15]. The thermal conductivities of encapsulation, substrate, stratum corneum, epidermis, dermis, and fat are 0.2 W/(m·K), 0.15 W/(m·K), 0.21 W/(m·K), 0.21 W/(m·K), 0.37 W/(m·K) and 0.16 W/(m·K) [4,15]. The top surface of encapsulation layer has a natural convection boundary with the coefficient of heat convection *h* = 25 W/(m^2^·K) [2,16]. The ambient temperature is set as 25 °C. The core body temperature at the bottom surface of substrate is 37 °C. The metabolic heat generation in the skin tissue is 368 W/m^3^ [15]. The product of mass density and specific heat capacity of the blood is 4.218 × 10^6^ J/(m^3^·K) [17]. The blood perfusion rate inside the dermis layer is 0.03 mL/(mL·s) [15]. Figure 2 shows the comparison of temperature increase along the thickness direction between the analytic predictions and FEA under the input power density 2500 W/m^2^. The good agreement validates the analytic model. With the distance to the top surface of encapsulation layer increasing, the temperature increase first increases inside the encapsulation layer then decreases in other layers. The temperature increase of the heat source can reach about 28 °C while the temperature increase at the device/skin interface is about 9 °C. Compared to the temperature increase of skin away from electronic device, which is about −1.5 °C, the constant input power causes a remarkable temperature increase.

## 3. Thermal Analysis under a Pulsed Power

As shown in Figure 2, the temperature increase of the device/skin interface is much more than that the human can stand. It may induce uncomfortable feelings or even tissue lesion in bio-integrated applications. Clearly, the temperature increase can be reduced by decreasing the input power density. However, the devices can hardly operate properly under small input power. In order to solve this dilemma, Kim et al. [6] and Li et al. [7] changed the input loading method from constant power to pulsed protocol, which has proven pretty effective on modern devices. The operation of flexible electronic devices (e.g., flexible light-emitting diodes) in a pulsed mode could significantly reduce the temperature increase to reach the goal in thermal management. In this section, an analytic model is developed to investigate the thermal response of flexible electronic devices on human skin.

**Figure 3:**
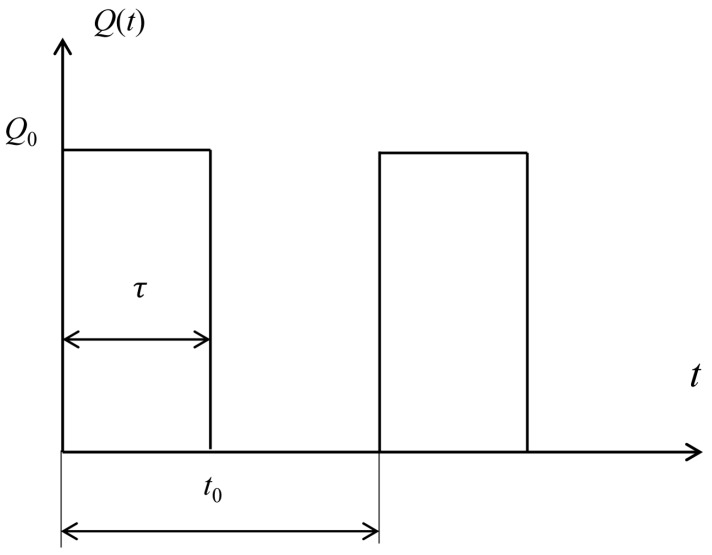
The pulsed power density *Q*(*t*) with *Q*_0_ as the peak power density, *t* as the during time and *t*_0_ as the period.

Under a pulsed power, the temperature in the system increases to saturation in a fluctuation way [12]. We are interested in the saturated temperature since it gives the maximum temperature that could reach in the system. The pulsed power *Q*(*t*) is defined in Figure 3 with *Q*_0_ as the peak value and *τ* as the heating duration in one period *t*_0_. The duty cycle *D* is given by *D* = *τ/t*_0_. Under a pulsed power density *Q*(*t*), the temperature increase from the core body temperature in the system satisfies,

(10) $$\left\{ \begin{array}{l} k_{i}\frac{\partial^{2}\Delta T_{i}}{\partial z^{2}}=\rho_{i}c_{i}\frac{\partial \Delta T_{i}}{\partial t}\;\;\;\;\;\;\;\;\;\;\left(i=1,2\right)\\ k_{i}\frac{\partial^{2}\Delta T_{i}}{\partial z^{2}}+q_{\mathrm{met}}=\rho_{i}c_{i}\frac{\partial \Delta T_{i}}{\partial t}\;\;\;\;\;\;\;\;\;\;\left(i=3,4,6\right)\\ k_{5}\frac{\partial^{2}\Delta T_{5}}{\partial z^{2}}-\varpi_{b}\rho_{b}c_{b}\Delta T_{5}+q_{\mathrm{met}}=\rho_{5}c_{5}\frac{\partial \Delta T_{5}}{\partial t} \end{array}\right.\;\;\;$$

where ρ*_i_* and *c_i_* are the mass density and the specific heat capacity for each layer. The boundary and continuity conditions are the same as those for the case under a constant power in Equations (5)–(8) except the constant power density *Q* in Equation (6) should be changed to *Q*(*t*), i.e.,

(11) $${\Delta T|}_{z=h_{1}^{+}}={\Delta T|}_{z=h_{1}^{-}}\;\text{and}\;k_{1}{\frac{\partial \Delta T}{\partial z}|}_{z=h_{1}^{-}}-k_{2}{\frac{\partial \Delta T}{\partial z}|}_{z=h_{1}^{+}}=Q\left(t\right)\;\;$$

The solution for Equation (10) is the summation of the specific solution, which is given in Section 2 without the input power, and the homogeneous solution, which is to be determined below. The homogenous solution for Equation (10) satisfies

(12) $$\left\{ \begin{array}{l} \frac{\partial \Delta T_{i}}{\partial t}-\;\lambda_{i}\frac{\partial^{2}\Delta T_{i}}{\partial z^{2}}\;=0\;\;\;\left(i=1,2,3,4,6\right)\\ \frac{\partial \Delta T_{5}}{\partial t}-\lambda_{5}\frac{\partial^{2}\Delta T_{5}}{\partial z^{2}}+\varpi_{b}\Delta T_{5}=0 \end{array}\right.\;\;\;$$

where λ*_i_* = *k_i_*/(*c_i_*ρ*_i_*) is the thermal diffusivity with *c*, ρ and *k* as the specific heat capacity, mass density and thermal conductivity, respectively.

The method of superposition is adopted to obtain the temperature increase after saturation (i.e., homogeneous solution) under a pulsed power density, which could be written via Fourier series by

(13) $$Q\left(t\right)=Q_{0}\{\begin{array}{cc} 1 & 0<t\leq \tau \\ 0 & \tau <t\leq t_{0} \end{array}=Q_{0}\left[a_{0}+\sum_{n=1}^{\infty }\left(a_{n}\cos n\omega t+b_{n}\sin n\omega t\right)\right]$$

where $\omega =2\pi /t_{0}$, $a_{0}=D=\tau /t_{0}$, $a_{n}=\sin\left(2n\pi D\right)/\left(n\pi \right)$, and $b_{n}=\frac{\left[1-\cos\left(2n\pi D\right)\right]}{n\pi }$. The temperature increase after saturation due to each sinusoidal power density $Q_{0}\cos n\omega t$ (or $Q_{0}\sin n\omega t$) in Equation (13) corresponds to the real (or imaginary part) of the solution due to a power of $Q_{0}e^{n\omega t\cdot i}$. The power of $Q_{0}e^{n\omega t\cdot i}$ yields the temperature increase as $\theta \left(z;n\omega \right)e^{n\omega t\cdot i}$, which gives the real and imaginary part as $\left|\theta \left(z;n\omega \right)\right|\cos\left(n\omega t+\beta_{n}\right)$ and $\left|\theta \left(z;n\omega \right)\right|\sin\left(n\omega t+\beta_{n}\right)$, respectively. Here $\beta_{n}\left(n\omega \right)$ is the phase angle of θ(z;nω). Therefore, the temperature increase due to the pulsed power can be obtained by

(14) $$\Delta T\left(z,t\right)=D\theta \left(z;0\right)+\sum_{n=1}^{\infty }\left|\theta \left(z;n\omega \right)\right|\cdot \left[\begin{array}{l} \frac{\sin\left(2n\pi D\right)}{n\pi }\cos\left(n\omega t+\beta_{n}\right)\\ +\frac{1-\cos\left(2n\pi D\right)}{n\pi }\sin\left(n\omega t+\beta_{n}\right) \end{array}\right]$$

where θ(z;nω) is to be determined from Equation (11) and boundary conditions. The substitution of $\theta \left(z;n\omega \right)e^{n\omega t\cdot i}$ into Equation (11) gives the governing equation of θ(z;nω) as

(15) $$\left\{ \begin{array}{l} \frac{d^{2}\theta_{i}}{dz^{2}}-q_{i}^{2}\theta_{i}\;=\;\;\;0\;\;\;\;\;\;\left(i=1,2,3,4,6\right)\\ \frac{d^{2}\theta_{5}}{dz^{2}}-\left(q_{5}^{2}+\eta /k_{5}\right)\theta_{5}\;=\;\;\;0\; \end{array}\right.$$

where the $q^{2}=n\omega i/\lambda$. θ(z;nω) satisfies the following boundary and continuity conditions: ${-k_{1}\partial \theta /\partial z|}_{z=0}=-h\cdot {\theta |}_{z=0}$, ${\theta |}_{z=h_{1}^{+}}={\theta |}_{z=h_{1}^{-}}$, $k_{1}{\partial \theta /\partial z|}_{z=h_{1}^{-}}-k_{2}{\partial \theta /\partial z|}_{z=h_{1}^{+}}=Q_{0}$, ${\theta |}_{z={\left(\sum_{i=1}^{n}h_{i}\right)}^{+}}={\theta |}_{z={\left(\sum_{i=1}^{n}h_{i}\right)}^{-}}$, $k_{\left(n+1\right)}{\partial \theta /\partial z|}_{z={\left(\sum_{i=1}^{n}h_{i}\right)}^{+}}=k_{n}{\partial \theta /\partial z|}_{z={\left(\sum_{i=1}^{n}h_{i}\right)}^{-}}$, ${\theta |}_{z=\sum_{i=1}^{6}h_{i}}=0$.

The solution of Equation (15) takes the form of

(16) $$\left\{ \begin{array}{l} \theta_{i}=A_{i}\exp\left(z\sqrt{q_{i}^{2}}\right)+B_{i}\exp\left(-z\sqrt{q_{i}^{2}}\right)\;\;\;\;\;\;\;\;\;\left(i=1,2,3,4,6\right)\\ \theta_{5}=A_{5}\exp\left(z\sqrt{q_{5}^{2}+\eta /k_{5}}\right)+B_{5}\exp\left(-z\sqrt{q_{5}^{2}+\eta /k_{5}}\right)\; \end{array}\right.$$

where the unknown coefficients in Equation (16) are to be determined by the boundary and continuity conditions as

(17) $$\left\{\begin{array}{l} A_{1}^{\prime }\\ B_{1}^{\prime }\\ A_{2}^{\prime }\\ B_{2}^{\prime }\\ A_{3}^{\prime }\\ B_{3}^{\prime }\\ A_{4}^{\prime }\\ B_{4}^{\prime }\\ A_{5}^{\prime }\\ B_{5}^{\prime }\\ A_{6}^{\prime }\\ B_{6}^{\prime } \end{array}\right\}=\left\{\begin{array}{l} \frac{T_{1}R\cos h\left(h_{6}\sqrt{q_{6}^{2}}\right)+m_{5}RT_{2}\sin h\left(h_{6}\sqrt{q_{6}^{2}}\right)}{\left(P_{1}S_{1}+m_{1}P_{2}T_{1}\right)\cos h\left(h_{6}\sqrt{q_{6}^{2}}\right)+m_{5}\left(P_{1}S_{2}+m_{1}P_{2}T_{2}\right)\sin h\left(h_{6}\sqrt{q_{6}^{2}}\right)}\\ \left(k_{1}\sqrt{q_{1}^{2}}-h\right)A_{1}/\left(k_{1}\sqrt{q_{1}^{2}}+h\right)\\ \left[\left(P_{1}+m_{1}P_{2}\right)A_{1}-R\right]e^{-h_{1}\sqrt{q_{2}^{2}}}\\ \left[\left(P_{1}-m_{1}P_{2}\right)A_{1}+R\right]e^{h_{1}\sqrt{q_{2}^{2}}}\\ \left(G+H\right)e^{-\left(h_{1}+h_{2}\right)\sqrt{q_{3}^{2}}}\\ \left(G-H\right)e^{\left(h_{1}+h_{2}\right)\sqrt{q_{3}^{2}}}\\ \left(G+H\right)e^{-\left(h_{1}+h_{2}\right)\sqrt{q_{3}^{2}}}\\ \left(G-H\right)e^{\left(h_{1}+h_{2}\right)\sqrt{q_{3}^{2}}}\\ \left(I+J\right)e^{-\left(h_{1}+h_{2}+h_{3}+h_{4}\right)\sqrt{q_{5}^{2}+\eta /k_{5}}}\\ \left(I-J\right)e^{\left(h_{1}+h_{2}+h_{3}+h_{4}\right)\sqrt{q_{5}^{2}+\eta /k_{5}}}\\ \left(K+L\right)e^{-\left(h_{1}+h_{2}+h_{3}+h_{4}+h_{5}\right)\sqrt{q_{6}^{2}}}\\ \left(K-L\right)e^{\left(h_{1}+h_{2}+h_{3}+h_{4}+h_{5}\right)\sqrt{q_{6}^{2}}} \end{array}\right\}$$

where

$$\begin{array}{l} P_{1}=\frac{k_{1}\sqrt{q_{1}^{2}}\cos h\left(h_{1}\sqrt{q_{1}^{2}}\right)+h\sin h\left(h_{1}\sqrt{q_{1}^{2}}\right)}{k_{1}\sqrt{q_{1}^{2}}+h}\\ P_{2}=\frac{k_{1}\sqrt{q_{1}^{2}}\sin h\left(h_{1}\sqrt{q_{1}^{2}}\right)+h\cos h\left(h_{1}\sqrt{q_{1}^{2}}\right)}{k_{1}\sqrt{q_{1}^{2}}+h}\\ R=\frac{Q_{0}}{2k_{2}\sqrt{q_{2}^{2}}} \end{array}$$

$$S_{1}=\left[\begin{array}{l} \cos h\left(h_{2}\sqrt{q_{2}^{2}}\right)\cos h\left(\left(h_{3}+h_{4}\right)\sqrt{q_{3}^{2}}\right)\cos h\left(h_{5}\sqrt{q_{5}^{2}+\eta /k_{5}}\right)\\ +m_{2}\sin h\left(h_{2}\sqrt{q_{2}^{2}}\right)\sin h\left(\left(h_{3}+h_{4}\right)\sqrt{q_{3}^{2}}\right)\cos h\left(h_{5}\sqrt{q_{5}^{2}+\eta /k_{5}}\right)\\ +m_{4}\cos h\left(h_{2}\sqrt{q_{2}^{2}}\right)\sin h\left(\left(h_{3}+h_{4}\right)\sqrt{q_{3}^{2}}\right)\sin h\left(h_{5}\sqrt{q_{5}^{2}+\eta /k_{5}}\right)\\ +m_{2}m_{4}\sin h\left(h_{2}\sqrt{q_{2}^{2}}\right)\cos h\left(\left(h_{3}+h_{4}\right)\sqrt{q_{3}^{2}}\right)\sin h\left(h_{5}\sqrt{q_{5}^{2}+\eta /k_{5}}\right) \end{array}\right]$$

$$T_{1}=\left[\begin{array}{l} \sin h\left(h_{2}\sqrt{q_{2}^{2}}\right)\cos h\left(\left(h_{3}+h_{4}\right)\sqrt{q_{3}^{2}}\right)\cos h\left(h_{5}\sqrt{q_{5}^{2}+\eta /k_{5}}\right)\\ +m_{2}\cos h\left(h_{2}\sqrt{q_{2}^{2}}\right)\sin h\left(\left(h_{3}+h_{4}\right)\sqrt{q_{3}^{2}}\right)\cos h\left(h_{5}\sqrt{q_{5}^{2}+\eta /k_{5}}\right)\\ +m_{4}\sin h\left(h_{2}\sqrt{q_{2}^{2}}\right)\sin h\left(\left(h_{3}+h_{4}\right)\sqrt{q_{3}^{2}}\right)\sin h\left(h_{5}\sqrt{q_{5}^{2}+\eta /k_{5}}\right)\\ +m_{2}m_{4}\cos h\left(h_{2}\sqrt{q_{2}^{2}}\right)\cos h\left(\left(h_{3}+h_{4}\right)\sqrt{q_{3}^{2}}\right)\sin h\left(h_{5}\sqrt{q_{5}^{2}+\eta /k_{5}}\right) \end{array}\right]$$

$$S_{2}=\left[\begin{array}{l} \cos h\left(h_{2}\sqrt{q_{2}^{2}}\right)\cos h\left(\left(h_{3}+h_{4}\right)\sqrt{q_{3}^{2}}\right)\sin h\left(h_{5}\sqrt{q_{5}^{2}+\eta /k_{5}}\right)\\ +m_{2}\sin h\left(h_{2}\sqrt{q_{2}^{2}}\right)\sin h\left(\left(h_{3}+h_{4}\right)\sqrt{q_{3}^{2}}\right)\sin h\left(h_{5}\sqrt{q_{5}^{2}+\eta /k_{5}}\right)\\ +m_{4}\cos h\left(h_{2}\sqrt{q_{2}^{2}}\right)\sin h\left(\left(h_{3}+h_{4}\right)\sqrt{q_{3}^{2}}\right)\cos h\left(h_{5}\sqrt{q_{5}^{2}+\eta /k_{5}}\right)\\ +m_{2}m_{4}\sin h\left(h_{2}\sqrt{q_{2}^{2}}\right)\cos h\left(\left(h_{3}+h_{4}\right)\sqrt{q_{3}^{2}}\right)\cos h\left(h_{5}\sqrt{q_{5}^{2}+\eta /k_{5}}\right) \end{array}\right]$$

$$T_{2}=\left[\begin{array}{l} \sin h\left(h_{2}\sqrt{q_{2}^{2}}\right)\cos h\left(\left(h_{3}+h_{4}\right)\sqrt{q_{3}^{2}}\right)\sin h\left(h_{5}\sqrt{q_{5}^{2}+\eta /k_{5}}\right)\\ +m_{2}\cos h\left(h_{2}\sqrt{q_{2}^{2}}\right)\sin h\left(\left(h_{3}+h_{4}\right)\sqrt{q_{3}^{2}}\right)\sin h\left(h_{5}\sqrt{q_{5}^{2}+\eta /k_{5}}\right)\\ +m_{4}\sin h\left(h_{2}\sqrt{q_{2}^{2}}\right)\sin h\left(\left(h_{3}+h_{4}\right)\sqrt{q_{3}^{2}}\right)\cos h\left(h_{5}\sqrt{q_{5}^{2}+\eta /k_{5}}\right)\\ +m_{2}m_{4}\cos h\left(h_{2}\sqrt{q_{2}^{2}}\right)\cos h\left(\left(h_{3}+h_{4}\right)\sqrt{q_{3}^{2}}\right)\cos h\left(h_{5}\sqrt{q_{5}^{2}+\eta /k_{5}}\right) \end{array}\right]$$

$$m_{i}=\frac{k_{i}\sqrt{q_{i}^{2}}}{k_{i+1}\sqrt{q_{i+1}^{2}}}\left(i=1,2,3\right)$$

$$\begin{array}{l} m_{4}=\frac{k_{4}\sqrt{q_{4}^{2}}}{k_{5}\sqrt{q_{5}^{2}+\eta /k_{5}}}\\ m_{5}=\frac{k_{5}\sqrt{q_{5}^{2}+\eta /k_{5}}}{k_{6}\sqrt{q_{6}^{2}}} \end{array}$$

$$G=\left[P_{1}\cos h\left(h_{2}\sqrt{q_{2}^{2}}\right)+m_{1}P_{2}\sin h\left(h_{2}\sqrt{q_{2}^{2}}\right)\right]A_{1}^{\prime }-R\sin h\left(h_{2}\sqrt{q_{2}^{2}}\right)$$

$$H=m_{2}\left\{\left[P_{1}\sin h\left(h_{2}\sqrt{q_{2}^{2}}\right)+m_{1}P_{2}\cos h\left(h_{2}\sqrt{q_{2}^{2}}\right)\right]A_{1}^{\prime }-R\cos h\left(h_{2}\sqrt{q_{2}^{2}}\right)\right\}$$

$$I=G\cos h\left[\left(h_{3}+h_{4}\right)\sqrt{q_{3}^{2}}\right]+H\sin h\left[\left(h_{3}+h_{4}\right)\sqrt{q_{3}^{2}}\right]$$

$$J=m_{4}\left\{G\sin h\left[\left(h_{3}+h_{4}\right)\sqrt{q_{3}^{2}}\right]+H\cos h\left[\left(h_{3}+h_{4}\right)\sqrt{q_{3}^{2}}\right]\right\}$$

$$K=I\cos h\left(h_{5}\sqrt{q_{5}^{2}+\eta /k_{5}}\right)+J\sin h\left(h_{5}\sqrt{q_{5}^{2}+\eta /k_{5}}\right)$$

$$L=m_{5}\left[I\sin h\left(h_{5}\sqrt{q_{5}^{2}+\eta /k_{5}}\right)+J\cos h\left(h_{5}\sqrt{q_{5}^{2}+\eta /k_{5}}\right)\right]$$

Thus, the temperature increase of heat source and device/skin interface can be given as follows,

(18) $$\begin{array}{ll} \Delta T_{\mathrm{device}}(t;\omega )= & \Delta T_{\mathrm{initial}}(h_{1})+D\theta_{1}(h_{1};0)\\ & +\sum_{n=1}^{\infty }\left|\theta_{1}(h_{1};n\omega )\right|\;\cdot \;\;\left[\begin{array}{l} \frac{\sin\left(2n\pi D\right)}{n\pi }\cos\left(n\omega t+\beta_{n}^{\mathrm{surface}}\right)\\ +\frac{1-\cos\left(2n\pi D\right)}{n\pi }\sin\left(n\omega t+\beta_{n}^{\mathrm{surface}}\right) \end{array}\right] \end{array}$$

(19) $$\begin{array}{ll} \Delta T_{\mathrm{interface}}(t;\omega )= & \Delta T_{\mathrm{initial}}(h_{1}+h_{2})+D\theta_{2}(h_{1}+h_{2};0)\\ & +\sum_{n=1}^{\infty }\left|\theta_{2}(h_{1}+h_{2};n\omega )\right|\;\cdot \;\;\;\left[\begin{array}{l} \frac{\sin\left(2n\pi D\right)}{n\pi }\cos\left(n\omega t+\beta_{n}^{\mathrm{surface}}\right)\\ +\frac{1-\cos\left(2n\pi D\right)}{n\pi }\sin\left(n\omega t+\beta_{n}^{\mathrm{surface}}\right) \end{array}\right] \end{array}$$

where Δ*T*_initial_ denotes the specific solution [initial temperature increase field in Equation (4) when there is no input power, given by

(20) $$\Delta T_{\mathrm{initial}}\left(h_{1}\right)=\left(\frac{k_{1}}{h}+h_{1}\right)A_{1}+\Delta T_{0}$$

and

(21) $$\Delta T_{\mathrm{initial}}\left(h_{1}+h_{2}\right)=\left(\frac{k_{1}}{h}+h_{1}+\frac{k_{1}h_{2}}{k_{2}}\right)A_{1}+\Delta T_{0}$$

Here $\theta_{1}(h_{1};n\omega )$ and $\theta_{2}(h_{1}+h_{2};n\omega )$ can be obtained from Equations (16) and (17) as

(22) $$\theta_{1}\left(h_{1};n\omega \right)=2\left[k_{1}\sqrt{q_{1}^{2}}\cos h\left(h_{1}\sqrt{q_{1}^{2}}\right)+h\sin h\left(h_{1}\sqrt{q_{1}^{2}}\right)\right]A_{1}^{\prime }/\left(k_{1}\sqrt{q_{1}^{2}}+h\right)$$

and

(23) $$\theta_{2}\left(h_{1}+h_{2};n\omega \right)=2\left[P_{1}A_{1}^{\prime }\cos h\left(-h_{2}\sqrt{q_{2}^{2}}\right)+\left(m_{1}P_{2}-R\right)\sin h\left(-h_{2}\sqrt{q_{2}^{2}}\right)\right]$$

To validate the analytic model under a pulsed power, we also performed FEA to obtain the temperature increase after saturation due to the pulsed power. The continuum element DC3D8 in ABAQUS is used to discretize the geometry. The material and geometry parameters such as thicknesses and thermal conductivity of each layer are the same as those in Section 2. The thermal diffusivity of encapsulation, substrate, stratum corneum, epidermis, dermis and fat are 1.4 × 10^−7^ m^2^/s, 1.1 × 10^−7^ m^2^/s, 6.6 × 10^−8^ m^2^/s, 6.6 × 10^−8^ m^2^/s, 1.3 ×10^−7^ m^2^/s, 8.1 × 10^−8^ m^2^/s, respectively [6,14]. The metabolic heat generation is set as 368 W/m^3^ [14]. The natural convection boundary with the coefficient of heat convection 25 W/(m^2^·K) is applied on the top surface of encapsulation layer. The ambient temperature is set as 25 °C. The core body temperature at the bottom surface of substrate is 37 °C. The thermal properties of blood and skin tissue can be found in Section 2 or in reference [14].

**Figure 4:**
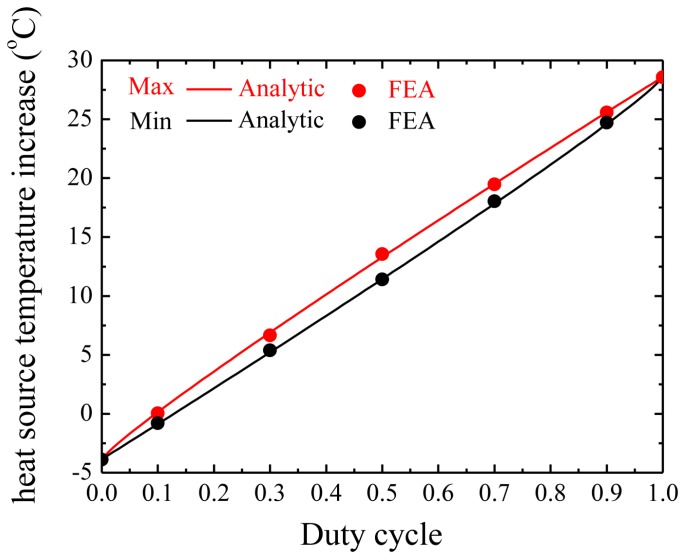
The maximum and minimum heat source temperature increase after saturation versus duty cycle.

Figure 4 shows the influence of duty cycle on the maximum and minimum heat source temperature increase under the pulsed peak power density of 2500 W/m^2^ (here we fix this peak power density to ensure that devices operate properly) with period as 500 ms. The analytic prediction agrees very well with FEA. Both the maximum temperature increase and minimum temperature increase of the heat source decrease as the duty cycle decreases. This trend can qualitatively be understood since the average emitted heat from the electronic device is reduced. For the case of constant power density corresponding to a duty cycle of 100%, the maximum temperature increase of heat source is 28.6 °C. As the duty cycle decreases to a smaller duty cycle of 10%, the maximum temperature increase of the heat source drops rapidly to about 0 °C. When the duty cycle is smaller than 10%, the temperature increase may become negative. This is because the temperature increase is defined from the core body temperature instead of the ambient temperature. A negative temperature increase means that the temperature is lower than the core body temperature.

It is necessary to mention that time period also has an influence on the results in a pulsed protocol. According to a transient heat transfer FEA, we obtained that the characteristic time of the temperature profile to reach a steady state is about 280 s. When the time periods used in the pulsed protocol are much smaller than the characteristic time, just as 500 ms we set above, the changes in time periods have only minor effects on the maximal heat source temperature. However, if the duration (*τ*) of the pulse is much longer than the characteristic time, the maximal heat source temperature is expected to be the same as if the electronic device is always turned on (constant protocol).

**Figure 5:**
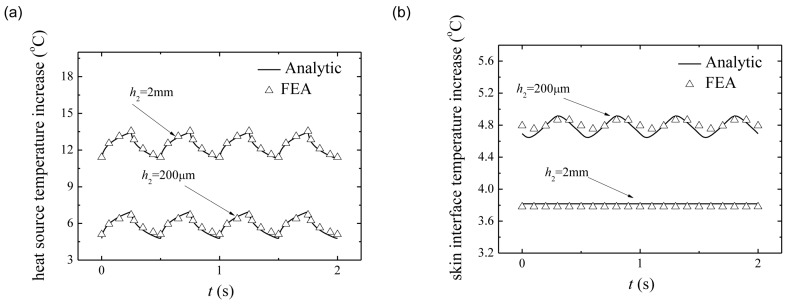
Temperature increase comparison between the analytic prediction and finite element analysis for the pulsed peak power density 2500 W/m^2^ with 50% duty cycle and period 500 ms: (**a**) heat source; and (**b**) skin surface.

Figure 5a compares the temperature increase of heat source after saturation from the analytic model in Equation (18) and FEA under the pulsed peak power density of 2500 W/m^2^ with duty cycle *D* as 50% and period as 500 ms. The good agreement between the analytical prediction and FEA validates the analytical model. The temperature increase of heat source decreases with the increase of substrate thickness. Figure 5b compares the temperature increase of skin surface after saturation versus time from the analytic model in Equation (19) and FEA under the same condition in Figure 5a. With the change of substrate thickness, the temperature increase of skin surface shows the opposite trend to Figure 5a, i.e., the temperature increase of skin surface increases with the increase of the substrate thickness. These results clearly show that the pulsed operation is very effective at reducing the temperature increase in the system. A thin substrate can reduce the device temperature increase due to the heat sink effect of skin while increase the temperature increase of skin surface due to the influence of heat source.

## 4. Conclusions

A one-dimensional analytic thermal model, as validated by the finite element analysis, for the flexible electronic devices integrated with human skin under a constant power and pulsed power is presented in this paper. This model combines the Fourier heat conduction equation for the flexible electronic devices and the Pennes bio-heat transfer equation for the skin tissue. The influences of geometric and loading parameters on the temperature increase under a pulsed power are investigated. The results could provide design guidelines for flexible electronic devices to minimize the adverse thermal effect in bio-integrated applications.
